# Suspicion and Treatment of Perilymphatic Fistula: A Prospective Clinical Study

**DOI:** 10.3390/audiolres14010006

**Published:** 2024-01-08

**Authors:** Issam Saliba, Naif Bawazeer, Sarah Belhassen

**Affiliations:** 1 Division of Otolaryngology-Head & Neck Surgery, University of Montreal, Montreal, QC H3C 3J7, Canada; 2 University of Montreal Hospital Centre Research Centre (CRCHUM), Montreal, QC H2X 0A9, Canada; 3 University of Montreal Hospital Centre (CHUM), Montreal, QC H2X 3E4, Canada

**Keywords:** perilymphatic fistula, hearing loss, fluctuating, dizziness, vertigo, perilymph, oval window, round window

## Abstract

***Background:*** Since the discovery of the perilymphatic fistula (PLF), the diagnosis and treatment remain controversial. If successfully recognized, the PLF is surgically repairable with an obliteration of the fistula site. Successful treatment has a major impact on patient’s quality of life with an improvement in their audiological and vestibular symptoms. ***Objective:*** To prospectively investigate patients’ clinical and audiological evolution with PLF suspicion after middle ear exploration and obliteration of the round and oval window. ***Study Design:*** Prospective comparative study. ***Setting:*** Tertiary care center. ***Methods:*** Patients were divided into two groups: Group I consisted of patients where no PLF had been identified intraoperatively at the oval and/or at the round window, and Group II consisted of patients where a fistula had been visualized. Patient assessment was a combination of past medical history, the presence of any risk factors, cochlear and vestibular symptoms, a physical examination, temporal bone imaging, audiograms, and a videonystagmogram (VNG). ***Results:*** A total of 98 patients were divided into two groups: 62 in Group I and 36 in Group II. A statistically significant difference regarding gender was observed in Group II (83.3% of males vs. 16.7% of females, *p* = 0.008). A total of 14 cases (4 and 10 in Groups I and II, respectively) were operated for a recurrent PLF. Fat graft material was used in the majority of their previous surgery; however, no difference was found when comparing fat to other materials. In addition, no statistically significant difference was noted between Groups I and II concerning predisposing factors, imaging, VNG, symptom evolution, or a physical exam before the surgery and at 12 months post-operative. However, both groups showed statistically significant hearing and vestibular improvement. On the other hand, the air conduction (AC) and bone conduction (BC) at each frequency were not statistically different between the two groups before surgery but showed statistically significant improvement at 12 months post-operatively, especially for the BC at the frequencies 250 (*p* = 0.02), 500 (*p* = 0.0008), and 1000 Hz (*p* = 0.04). ***Conclusions:*** Whenever you suspect a perilymphatic fistula, do not hesitate to explore middle ear and do window obliterations using a tragal perichondrium material. Our data showed that cochlear and vestibular symptoms improved whether a fistula had been identified or not.

## 1. Introduction

The perilymphatic fistula (PLF) is an abnormal leak of perilymphatic fluid from the inner ear to the middle ear. Historically, Gelle discovered that a manipulation of the stapes could cause vertigo in Ménière’s patients [1]. Hennebert, in 1905 [2], found that alternating positive and negative pressure in the ear canal of a syphilitic patient could cause vertigo, and the Hennebert test was initially believed to be associated with otological syphilis. However, Barany associated these findings with the hypermobility of the stapes [3]. In 1968, Fee et al. [4] first described the concept of a traumatic PLF in patients with normal ears after a head injury; in 1970, Stroud and Calcettera [5] described the concept of a spontaneous perilymph fistula. However, since its discovery, the diagnosis and treatment remain controversial. Many etiologic factors have been described, such as direct head trauma, barotrauma, acoustic trauma, or as a complication of a stapes surgery [3].

If successfully recognized, the PLF is surgically repairable with an obliteration of the fistula site. Successful treatment has a major impact on patient’s quality of life, with an improvement in their audiological and vestibular symptoms. However, as the volume of the perilymph is approximately 75 microliters [3], the diagnosis of a PLF remains a challenge for the clinician. Its small volume makes the operative visualization of the PLF difficult, and as a consequence, the volume collectible for the chemical test is unpredictable. Some techniques have been described to enhance the visualization of the PLF, such as prolonged microscopic examination, and if performed under general anesthesia, having the anesthetist increase the intrathoracic pressure [3]. Other methods that have been described for the identification of the PLF include audiometry, vestibular evoked myogenic potential (VEMP), electrocochleography (ECoG), imaging modalities, or even a videonystagmogram (VNG). However, none of the aforementioned tests are specific in diagnosing the PLF [6], and the visualization of perilymphatic fluid remains subjective. Some authors also proposed medical therapy with loop diuretics prior to surgery and exploring the middle ear in the management of the PLF [7].

Besides the Cochlin–Tomoprotein detection [6], exploratory surgery is currently the only confirmatory method. In 2015, our group published a paper to identify factors that could better predict the diagnosis of the perilymphatic fistula [8]. This retrospective chart review of 71 patients failed to identify factors that could better predict the diagnosis of the PLF. However, it showed that middle ear exploration with oval and round window obliteration is effective in treating PLF, especially in decreasing vestibular symptoms, even when the fistula is not identified intraoperatively. Since our study consisted of a retrospective chart review, we decided to perform a prospective clinical trial with the hypothesis of symptom improvement after oval and round windows obliteration, whenever we suspect the PLF, irrespective of the identification or not of the perilymph leak.

Thus, the objective of this study is to prospectively investigate the clinical and audiological evolution of patients with a PLF suspicion after middle ear exploration and obliteration of the round and oval window.

## 2. Methods

We performed a prospective clinical study from 2013 to 2019 in our tertiary care center, including patients with a suspicion of a PLF. All patients were operated by the senior author (I.S). Patients were then divided into two groups: Group I consisted of patients in whom no perilymphatic fistula had been identified intraoperatively at the oval and/or at the round window, and patients were classified as Group II if a fistula had been visualized in one or both windows.

This study was approved by our institutional review board and ethical committee. Informed consent was obtained from all subjects involved in the study.

### 2.1. Pre-Operative Clinical Data

The initial evaluation of patients was a combination of their past medical history, a physical examination, temporal bone imaging, audiograms, and a VNG. A checklist sheet for the PLF was followed to collect all data and perform the requested tests systematically. We collected past medical history and the presence of any risk factors, such as barotrauma, a head injury or penetrating trauma, acoustic trauma, and the history of stapes surgery (stapedotomy or stapedectomy). The presence of hypoacusis (stable, fluctuating, or sudden hearing loss), aural fullness, tinnitus, vertigo, instability, the sensation of falling, the Tullio phenomenon, and the presence of nausea and vomiting were recorded. We performed a complete otolaryngological physical exam with an emphasis on vestibular testing. It included the presence of spontaneous nystagmus, a pneumoscopy, Valsalva-induced nystagmus, the Hennebert sign, the Romberg test, the head-shake test, the clinical head impulse test, and the Fukuda test.

The dizziness handicap inventory (DHI) was not adopted as a parameter to express the patient’s subjective conditions. However, we used the direct clinical symptoms to evaluate dizziness and vertigo.

### 2.2. Pre-Operative Paraclinical Tests

All patients had an audiogram performed at the initial presentation. The air and bone conductions were tested at 250, 500, 1000, 2000, 3000, and 4000 Hz, and the hearing loss was classified as sensorineural, conductive, or mixed. A speech discrimination score (SDS) was also tested for all patients. Patients also had either a high-resolution computerized tomography (CT) scan or magnetic resonance imaging (MRI) of the temporal bone depending on the clinical manifestations to rule out a pneumolabyrinth, a displaced prosthesis in patients with a history of stapes surgery, a visible fracture line, or another lesion that could explain the patient’s symptomatology. A VNG was also performed to complete the investigation in patients presenting vestibular symptoms.

### 2.3. Intraoperative Data

All PLF surgeries were performed under local anesthesia as an office-based surgery. The presence or absence of a visible PLF on the round or oval window was described. The material used for the obliteration, either fat, perichondrium, or a combination of both, was also recorded. The tissue used for the obliteration was then covered by a few pieces of gel foam.

### 2.4. Post-Operative Information

At the first visit, which took place 1 week post-operatively, the external auditory canal was cleaned, and the tympanic membrane was identified. Patients were followed 1, 6, and 12 months after the surgery. A complete audiogram was performed at each evaluation, and all clinical symptoms and physical examination findings were recorded, as listed above.

### 2.5. Statistical Analysis

Chi-square tests were used to analyze the demographic data, the predisposing factors, the paraclinical tests, and the operative findings for both groups. The chi-square test was also used to describe symptom evolution with time within Groups I and II. A Fisher exact test was used for recurrent case analysis. A *p*-value less than 0.05 was considered statistically significant.

## 3. Results

### 3.1. Pre-Operative Data

This study included 98 patients divided into two groups: 62 in Group I (no identified PLF) and 36 in Group II (identified PLF). The mean age was 44.7 ± 14.20 in Group I and 49.9 ± 11.41 in Group II. No statistical difference regarding the age, the affected side, the bilaterality, or the recurrence of the disease was noted. However, a statistical difference regarding the gender was observed, with a higher percentage of a PLF in males in comparison to females in Group II (83.3% of males vs. 16.7% of females, *p* = 0.008) and statistically significant more females in Group I (*p* = 0.001). A total of 14 cases (4 from Group I and 10 from Group II) were operated for a recurrent PLF. Even though the number is largely different between males and females in Group II, no predictive factor for the PLF was found to be statistically significant (*p* = 0.2). All demographic data are reported in Table 1.

The presence of any predisposing factor to a PLF, such as a barotrauma, penetrating trauma, head trauma, or a history of barotrauma, was recorded. No statistical difference was noted between Groups I and II (Table 2).

### 3.2. Paraclinical Data and Intraoperative Findings

**Figure 1:**
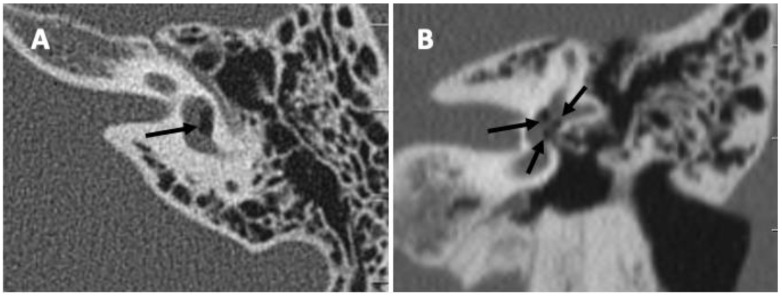
CT scan showing the pneumolabyrinth (arrows) on axial (**A**) and coronal (**B**) cuts.

A CT scan or an MRI was obtained depending on the clinical presentation, looking for a pneumolabyrinth, a visible fracture line, a displaced stapedial prosthesis, or another possible cause that could explain the patient’s symptoms. The differences between the two groups in finding a pneumolabyrinth on the pre-operative CT and MRI were not statistically significant (*p* = 0.23 and *p* = 0.74, respectively). Only two patients in each group presented with a pneumolabyrinth (Table 3). Figure 1 shows an example of a pneumolabyrinth on a CT scan.

### 3.3. Recurrent Cases

A total of 4 and 10 recurrent PLF cases were noted in Groups I and II, respectively, without a statistically significant difference (*p* = 0.3). In 100% of recurrent cases, fat material was used for the obliteration in Group I. In Group II, 60% fat, 20% perichondrium, and 20% fat and perichondrium material were used for obliteration. In the recurrent cases, no difference was found concerning the material used for window obliteration (*p* = 0.08).

Of the 36 patients included in Group II, where a PLF was identified during middle ear exploration, 22 were localized to the oval window, 12 were localized to the round window, and 2 had a PLF identified in both windows. Obliterations were performed on patients with a small piece of a cervical fat graft, a tragal perichondrium, or a combination of both. Both windows were obliterated in most of the patients, although the PLF was not identified. Pre-operatively, hearing was more affected in the group of the oval window fistula (PTA: 61 dB; SDS: 62%) than the round window fistula (PTA: 36 dB; SDS: 89%). The *p*-values were 0.04 and 0.03 for the PTA and SDS, respectively. Even though both patients’ fistulas (oval and round) were improved post-operatively, hearing remained more affected in the group of the oval window fistula (PTA: 45 dB; SDS: 56%) than the round window fistula (PTA: 25 dB; SDS: 81%). The *p*-values were 0.04 and 0.08 for the PTA and SDS, respectively. However, four patients in Group II had obliteration only of the oval window because two of them had a history of stapes surgery, and the other two had a history of barotrauma with a clear visualization of a PLF only at the oval window niche.

### 3.4. Videonystagmogram

Even though we aimed in this study to achieve a VNG for all patients presenting vestibular symptoms, we were unable to undertake the test for all patients in the short time between the suspicion of a PLF and the date of the surgery. Therefore, 34 patients from Group I and 22 from Group II achieved the VNG test pre-operatively. A VNG was not repeated post-operatively for ethical consideration, given that the test is unpleasant and makes the patient suffer from provoked vertigo. A total of 52.9% and 36.4% of patients showed caloric paresis on the affected side in Groups I and II. A caloric paresis above 25% was considered positive.

The mean paresis ± the standard deviation was 29.85% ± 26.89 and 27.18% ± 32.13 in Groups I and II, respectively. No statistically significant difference between the groups was noted (*p* = 0.55).

### 3.5. Audiometry—Bone Conductions Thresholds

**Figure 2:**
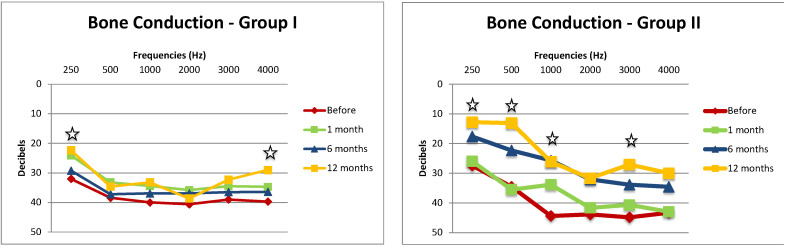
Bone conduction (BC) in Group I and Group II before the surgery, at 1, 6, and 12 months after. The star means the difference in BC is statistically significant between hearing before surgery and hearing at 12 months post-operatively.

We compared a bone conduction (BC) evolution at the frequencies of 250, 500, 1000, 2000, 3000, and 4000 Hz in Group I and in Group II before surgery and 1, 6, and 12 months post-operatively. (Figure 2). In Group I, when comparing the pre-operative BC to BC at 12 months post-operatively, the improvement in BC was statistically significant at frequencies of 250 (*p* = 0.03) and 4000 Hz (*p* = 0.04). In Group II, the decrease in BC was shown to be statistically significant at frequencies of 250 (*p* = 0.03), 500 (*p* < 0.0001), 1000 (*p* = 0.04), and 3000 Hz (*p* = 0.04).

**Figure 3:**
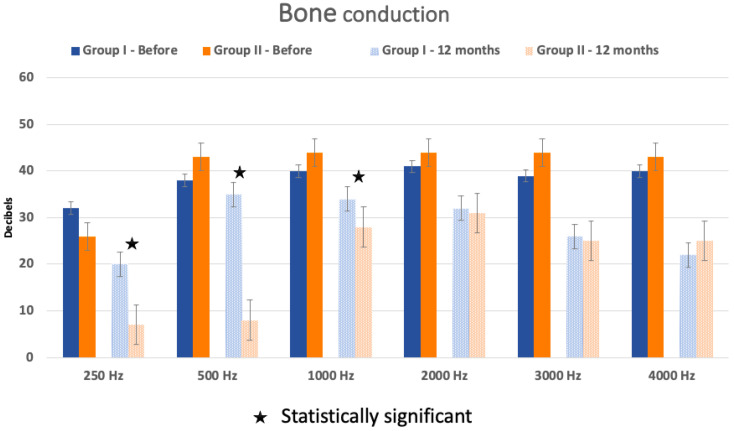
Bone conduction (BC) difference between Group I and Group II before the surgery and 12 months after. The star means the difference in BC is statistically significant between BC hearing before surgery and BC hearing at 12 months post-operatively. Standard errors are represented by the bars at the top of each bar chart.

On the other hand, the BC at each frequency was not statistically different between the two groups before surgery but significant at 12 months post-operatively at the frequencies 250 (*p* = 0.02), 500 (*p* = 0.0008), and 1000 Hz (*p* = 0.04) (Figure 3).

### 3.6. Audiometry—Air Conductions Thresholds

**Figure 4:**
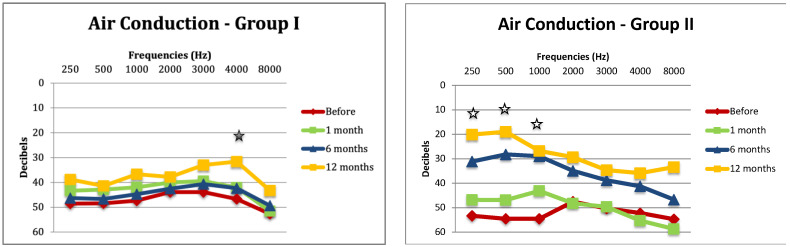
Air conduction (AC) in Group I and Group II before the surgery and 1, 6, and 12 months after. The star means the difference in AC is statistically significant between hearing before surgery and hearing at 12 months post-operatively.

An air conduction analysis showed a statistically significant improvement at the 4000 Hz frequency (*p* = 0.02) in Group I and at frequencies of 250 (*p* < 0.002), 500 (*p* < 0.0034), and 1000 Hz (*p* = 0.017) in Group II (Figure 4).

**Figure 5:**
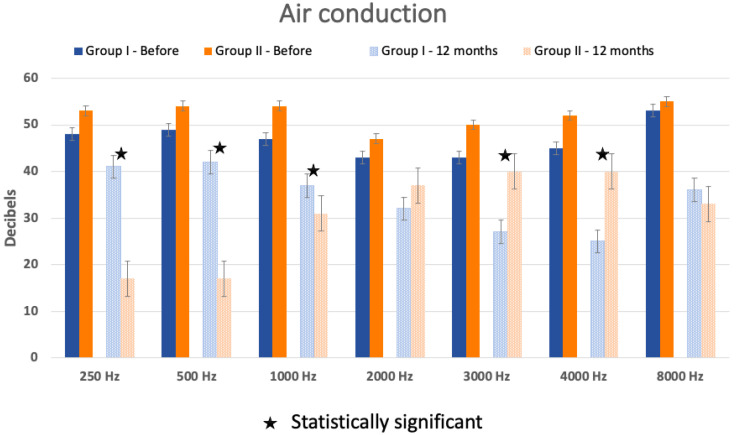
Air conduction (AC) difference between Group I and Group II before the surgery and 12 months after. The star means the difference in AC is statistically significant between AC hearing before surgery and AC hearing 12 months post-operatively. Standard errors are represented by the bars at the top of each bar chart.

On the other hand, the AC was not statistically different between the two groups before surgery but significant at 12 months post-operatively at the frequencies 250 (*p* = 0.03), 500 (*p* = 0.02), 1000 (*p* = 0.04), 3000 (*p* = 0.04), and 4000 Hz (*p* = 0.03) (Figure 5).

### 3.7. Audiometry—Speech Discrimination Score (SDS)

Single-syllable words in a list of 25 words are presented to the patient via earphones at a fixed, comfortable intensity level in a quiet sound room. The SDS for each ear is calculated from the percentage of words the patient repeats correctly.

**Figure 6:**
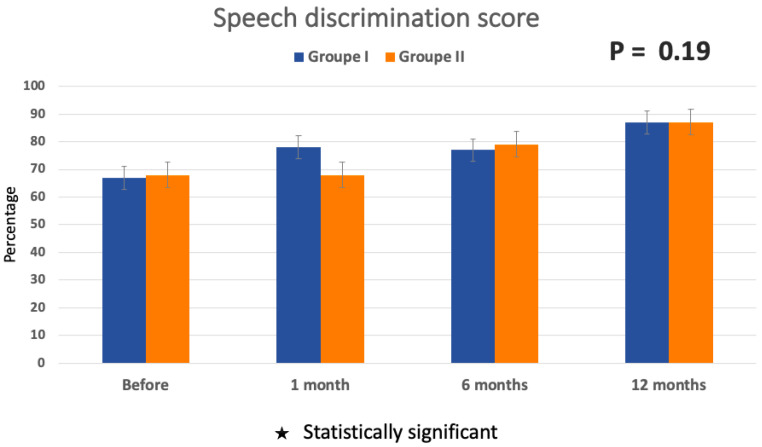
Speech discrimination score mean before and 12 months after surgery for Group I and Group II. Standard errors are represented by the bars at the top of each bar chart.

The analysis of the speech discrimination score in both Groups I and II before window obliteration (67.74 ± 36.81% and 67.78 ± 36.43%, respectively) and at 1 month, 6 months, and 12 months (87.14 ± 17.58% and 86.67 ± 16.65%, respectively, for Groups I and II) after surgery did not show improvement to be statistically significant (*p* = 0.19) (Figure 6).

### 3.8. Sudden Hearing Loss Subgroup

In the subgroup of patients presented with sudden hearing loss (SHL), the difference in the hearing loss level in Group II was statistically significant before and after surgery (61 dB vs. 39.4 dB, respectively; *p* = 0.0004); in addition, hearing loss in the subgroup presented with SHL was statistically more important than patients with no SHL in Group II (*p* = 0.004) and Group I (*p* = 0.0007). No difference was found when analyzing the round and oval fistula locations.

### 3.9. Clinical Findings: Symptoms and Physical Examination

Patients were carefully questioned at every visit, before the obliteration, and at 1 month, 6 months, and 12 months after by filling out the checklist sheet (Table 4).

All the clinical findings and evolution data were recorded at every visit (Table 4). At the analysis of the symptoms’ evolution, no statistical difference was seen between Group I and Group II before the surgery except for the instability at rest. However, 12 months after the obliteration, both groups were highly improved and presented similar findings. Furthermore, no difference was noted between the groups when analyzing the physical findings before and after the surgery.

### 3.10. Symptom Evolution within the Groups

The symptom evolution was analyzed within Group I, where no PLF was visualized, and within Group II, where a PLF was seen intraoperatively.

In both groups, we noted an important reduction in the audiological and vestibular symptoms. In Group I, a reduction of 87% of hypoacousis was seen (*p* = 0.005), vertigo was resolved in 100% of patients (*p* = 0.0006), and the sensation of aural fullness improved by 90% at 12 months (*p* = 0.0042). However, although tinnitus and the sensation of falling presented an improvement post-operatively, they did not show statistical significance (Table 5).

In Group II, a reduction of 87.5% of hypoacusis was seen (*p* = 0.009), vertigo was resolved in 100% of patients (*p* = 0.001), and the sensation of aural fullness improved by 100% at 12 months (*p* = 0.01). However, although tinnitus and the sensation of falling presented an improvement post-operatively, they did not show statistical significance (Table 5).

The vertigo exhibited a reduction of 100% by one year in Group II (*p* = 0.001). Although they did not show statistical significance, an important reduction in the other clinical symptoms was seen after the surgery (Table 5).

## 4. Discussion

A perilymphatic fistula (PLF), also known as a labyrinthine fistula, is defined as an abnormal communication between the perilymphatic space of the inner ear and the middle ear cavity. It usually results in a leakage of perilymphatic fluid into the middle ear through the round window, oval window, or both [9]. PLFs are classified as either congenital [10], as seen with congenital abnormalities of the temporal bone, or acquired [9]. Possible causes of an acquired PLF include physical trauma, barotrauma, or following stapes surgery as examples. The term spontaneous PLF is used in cases where a leak occurs following a vigorous Valsalva maneuver or a sudden increase in intracranial pressure [3]. Goodhill et al. [11] proposed that an implosive force, described as being an increase in middle ear pressure or explosive force, which is an increased pressure in the perilymph compartment, can lead to membranous ruptures and induce a perilymphatic fistula.

The PLF remains a diagnostic challenge for clinicians, as it may present with various symptoms of vertigo, hearing loss, and tinnitus [9]. In order to develop a better understanding of this entity, in 2014, our group reviewed seventy-one available patient files operated for suspicion of a PLF between 1983 and 2012 in our tertiary care center, trying to identify factors that could better predict the diagnosis [8]. Although this study failed to identify any factor, it showed that a middle ear exploration with obliteration of both windows effectively decreased vestibular symptoms of the visualization of a PLF. Furthermore, we suggest obliterating both windows when a PLF is suspected in the absence of another diagnosis. In the present study, we performed a prospective analysis of the clinical and audiological evolution of patients with a PLF suspicion, after middle ear exploration and obliteration of the round and oval window, even in the absence of an identified fistula. Our hypothesis is based on our previous study: if we suspect a PLF, symptoms will improve after oval and round window obliteration using a caulking mechanism of the leak site regardless of an identification or not of the perilymph leak.

### 4.1. Predisposing Factors

When comparing Groups I (fistula not identified) and II (fistula identified), no difference was seen regarding any predisposing factors to a PLF, such as a head or acoustic trauma, barotrauma, or a history of stapes surgery. Furthermore, no difference was seen regarding the initial imaging findings. Similar results were found in our previous retrospective study, even though trauma as a predisposing factor was present in 50.6% of the patients (head trauma: 19.7%, barotrauma: 23.9%, penetrating trauma: 4.2%, and acoustic trauma: 2.8%), no statistical difference was found between the two groups, with or without an identified fistula [7].

In a retrospective review published by Fitzgerald et al. [12], the authors studied 197 suspected PLFs who underwent a unilateral PLF repair. Only 28% of patients had a leak identified at the time of the surgery. However, 87% of patients improved whether a fistula was identified or not after obliteration of both windows. A study by Seltzer et al. [13] observed similar findings, with elimination or improvement in vestibular symptoms and stabilization or improvement in hearing after closure of the PLF. Our study supports the previous data available in the literature: 36.73% of suspected fistula were positive for PLF; however, we noted an improvement in both groups.

### 4.2. Hearing Test

The audiometry analysis showed an improvement in air and bone conduction in both groups, although not all frequencies were shown to be significant. Before surgery, AC and BC showed no difference between the two groups. However, this difference became statistically significant at 12 months post-operatively, showing improved AC and BC in the group where the PLF was identified. When the leaking is substantial, it is easier to identify, and therefore, sealing the fistula showed a better improvement in hearing loss, as we noticed in Group II. When the fissure is small, this can explain why the perilymphatic leak is very weak and even sometimes unidentified, so the improvement in hearing is not as significant as in cases where the flow is more important. Even though the speech discrimination score was improved clinically (more than 20% improvement in the word recognition score), no statistically significant difference was noted when comparing the SDS before and after the surgery.

### 4.3. Sudden SHL

Heilen et al. treated ninety patients with sudden SHL using exploratory tympanotomy and the sealing of the oval and round windows [14]; 10 patients (11 percent) were identified with a perilymphatic fistula, corresponding to the results obtained from their review (13.6 percent). In two cases only, the pre- and post-operative audiological evaluation results showed a significant hearing improvement after treatment. They concluded the absence of significant improvement after an exploratory tympanotomy and the sealing of the membranes for patients with a definite perilymphatic fistula presented with SHL [14]. Our findings were the opposite. Patients with SHL showed a clinical and statistical improvement in the two groups. This discrepancy could be explained by the difference in the inclusion criteria. We operated on patients where we suspected a PLF, not only because of SHL, whereas in their series, they operated on all SHL cases regardless of the associated symptoms.

### 4.4. Vestibular Symptoms

Furthermore, we noted an improvement in most symptoms in both groups, and vertigo was resolved completely. Nevertheless, a fistula has been identified intraoperatively or not. Instability at rest before the surgery was more frequent in the group with no identified fistula (*p* = 0.03); however, the caloric paresis was not statistically different between the two groups, reflecting no labyrinthine damage yet.

In our series, we emphasize the immediate relief of vestibular symptoms following PLF repair surgery, as reported by Matsuda et al. [15]. Smith and colleagues performed a detailed anatomical study and provided a comprehensive description of the intricate structure of the membranous labyrinth in 2021 [16]. The utricular macula is attached to the bony labyrinth by a perforated membrane, the membrana limitans, giving it some mobility. It is plausible that abnormal endolymph flow due to a window leak may magnify utricular mobility, leading to aberrant vestibulospinal signals that result in unilateral postural instability [17,18].

### 4.5. Fistula Leak Identification

The visual identification of a fluid leak is an objective and observer-dependent finding and may be influenced by a wide variety of factors, such as a Trendelenburg position or forced or sustained inspiration [14]. Furthermore, it may be attributable to a tiny leak [19], intermittent leaks, or a spontaneous healing leak. Therefore, although no leakage of perilymphatic fluid is seen, a diagnosis of a PLF may be made [20].

### 4.6. PLF Recurrence

Recurrence of a PLF was more pronounced in Group II (27%) without a statistically significant difference with Group I (6.5%). In most of the recurrent PLF cases, middle ear windows were obliterated using a fat graft material in their previous surgery, alone or with a perichondrium. Even though no statistical difference was found concerning the material used for window obliteration (*p* = 0.08), we stopped using fat grafts for PLF treatment. Our preferred material is the tragal perichondrium.

### 4.7. Diagnostic Tools

The perilymphatic fistula can be difficult to diagnose differentially prior to exploratory surgery. Numerous efforts to enhance the diagnosis of the PLF have been made.

#### 4.7.1. Audiology

Fraser et al. [21] established an audiometric test for the PLF. This test aims to improve the patient’s hearing with a suspected PLF by placing the patient in a supine position with the affected ear uppermost long enough to allow the cochlea to refill with perilymph. Postural audiometry was compared favorably in this small series with otoscopy and a vestibular examination to diagnose a PLF. However, this test did not gain popularity.

The lowered VEMP intensity and the increased amplitude, similar to superior canal dehiscence syndrome, may be found in the presence of a labyrinthine fistula because of increased inner ear immittance [22]. It is important to note the presence of common symptoms between a PLF and superior canal dehiscence syndrome (SCDS); therefore, if there is a high suspicion of SCDS, a high-resolution CT scan of the mastoid and VEMP test should be performed. On the other hand, in the perilymphatic fistula subjects, ECoG showed an elevated SP/AP ratio and a greater change in the SP/AP, particularly for the 6000 Hz tone bursts, with the subject in an upright position after lying in a horizontal position for 30 min with the test ear up and after 15 min with the test ear down [23,24]. However, the literature is very poor, with valid scientific data for a pre-operative PLF diagnosis using VEMP or ECoG.

#### 4.7.2. Imaging

Morris et al. [25] demonstrated that an MRI with contrast was a valuable examination modality for identifying a PLF in humans. However, in the presence of middle ear effusion, neither the CT scan nor the MRI can distinguish between the perilymphatic fluid or the middle ear secretions [26]. In addition, even in the absence of middle ear effusion, neither radiologic test can identify the tiny volume of the perilymphatic leak. Only two cases of a pneumolabyrinth were identified pre-operatively in Group II. Dubrulle et al. reported that a delayed postcontrast 3D-FLAIR MRI may reveal the perilymphatic fistula in patients with probable Ménière’s disease using the round window sign. This visual sign, defined as a nodular FLAIR high signal in the round window, is more sensitive than a temporal bone CT scan examination in detecting PLF [27].

On the other hand, Venkatasamy et al. reported that the most common sign of a PLF on imaging CT + MRI (T2W SSFP without contrast) prior to surgery is the presence of a fluid filling in the RW (especially if > 2/3 of the RW niche) or the OW niches on both the CT and MRI [28]. Experimental studies show that the accumulation of perilymph was visualized in a guinea pig model of a PLF after an intravenous injection of Gd-DTPA-BMA [29]; in addition, the PLF may also modify the uptake dynamics of Gd-DTPA-BMA after intratympanic delivery [30].

#### 4.7.3. PLF Diagnosis Scale

Bussières et al. reported in 2003 a diagnosis scale to evaluate the risk of a PLF based on clinical situations, physical exams, and complementary exams to help the clinician evaluate a PLF. Thus, they consider that a patient with a score of 7 or more will have a high probability of having a PLF, in which case surgical exploration is recommended. For a score between 4 and 6, it is necessary to carefully evaluate the clinical presentation as a whole and decide whether the tympanotomy explorer is required. Finally, a score less than 4 excludes the diagnosis of a PLF [31]. In 2020, Sarna et al. similarly proposed a set of diagnostic criteria to help identify a definite and probable PLF [32].

#### 4.7.4. Cranio-Corpo-Graphy Test (CCG)

The CCG is a stepping test in which the movements of the head and shoulders are recorded. When turning to the lesion site, disequilibrium was considered suggestive of a PLF [33,34]. Voorhees et al. [35] studied the role of dynamic posturography in a neurotologic diagnosis. It showed that 64% of patients suspected of a PLF presented an abnormal result with this modality test.

#### 4.7.5. Dilute Fluorescein

Aksoy et al. reported in 2020 the advantage of a topical application of dilute fluorescein in diagnosing and localizing a perilymphatic fistula. A clear change in color was distinguished from yellow to green, leading to a diagnosis of the perilymphatic fistula and showing the origin of the fistula [36]. However, it remains an intraoperative test, which does not help in the pre-operative diagnosis.

#### 4.7.6. Cochlin–Tomoprotein

Finally, the role of the Cochlin–Tomoprotein (CTP) detection test has been proposed by Ikezono et al. for the diagnosis of a PLF [6,15]. As it has been shown to have a high specificity for perilymph detection, future research may show an increasing role of CTP in diagnosing a PLF. Using a perilymph-specific CTP as a diagnostic biomarker could prove that the PLF could be responsible for the disequilibrium and related auditory disturbances, especially in patients where the fistula is not identified intraoperatively.

### 4.8. Hypermobile Stapes Footplate

A previously underappreciated membranous or hypermobile stapes footplate can occur following head trauma and can cause intractable dizziness like PLF symptoms or third window syndrome. High-resolution temporal bone CT scans using the gray-scale inversion (invert) function can assist in the pre-operative diagnosis of a hypermobile stapes footplate [37]. It is one of the differential diagnoses of a PLF. Durable long-term success can be achieved by utilizing a fat graft patching of the round and oval windows in the same way as a PLF is treated. A major question to ask: Are we underdiagnosing the hypermobile stape footplate and mixing up the diagnosis with the unidentified PLF, as in our Group I? A prospective study with a large series could be performed to answer this question.

### 4.9. To Obliterate or Not to Obliterate Oval and Round Windows

The variability in the clinical presentation and the rarity of a positive physical exam make diagnosing a PLF more challenging for the otolaryngologist. The management of a PLF is still controversial; spontaneous healing may be achieved with bed rest. On the other hand, there is a risk of deterioration of the symptoms associated with the endolymphatic compartment collapsing. Early exploration may thus be advocated. In our data, we found some patients suffering only from dizziness or only from fluctuating hearing loss, making the PLF presentation atypical and the diagnosis very tricky. In the absence of a very clear diagnosis, when other differential diagnoses of the PLF are ruled out, and since the consequences of an untreated PLF are major, like debilitating vertigo and permanent hearing loss compared to the minor or absent surgical complications, we suggest the obliteration of both inner ear windows in all cases where a PLF is suspected. The surgical technique is quick and could be easily performed in an office-based setting.

## 5. Conclusions

The diagnosis of PLF remains challenging for the otolaryngologist, as its clinical presentation may vary significantly. Our prospective study confirms our retrospective findings: whenever you suspect a perilymphatic fistula, do not hesitate to perform the middle ear exploration and both window obliterations using a tragal perichondrium. Our data showed improvement in cochlear and vestibular symptoms, whether a fistula has been identified or not. We perform the PLF procedure under local anesthesia in an office-based setting. The benefit of the surgery outlines the risks of permanent hearing loss and debilitating vertigo.

## Figures and Tables

**Table 1:** Demographic data of patients in Group I and Group II.

		Group I (No PLF Identified) (N = 62)	Group II (PLF Identified) (N = 36)	*p-*Value
**Age (Years** **± SD)**		44.7 ± 14.20	49.9 ± 11.41	*p* = 0.21
**Gender**	Female	34 (54.8%)	6 (16.7%)	*p* = 0.001
	Male	28 (45.2%)	30 (83.3%)	
**Side**	Right	26 (41.9%)	22 (61.1%)	*p* = 0.20
	Left	36 (58.1%)	14 (38.9%)	
**Bilateral disease**		2 (3.23%)	0 (0%)	N/A
**Recurrence**		4 (6.5%)	10 (27.8%)	*p* = 0.08

**Table 2:** Predisposing factors to a perilymphatic fistula. N/A: not applicable.

	Group I (No PLF Identified) (N = 62)	Group II (PLF Identified) (N = 36)	*p*-Value
**Barotrauma**	n = 4 (6.5%)	n = 10 (29.4%)	*p* = 0.08
**Head trauma**	n = 6 (9.7%)	n = 2 (5.9%)	*p* = 1.00
**Acoustic trauma**	n = 0 (0%)	n = 2 (5.9%)	*p* = 0.35
**Penetrating trauma**	n = 0 (0%)	n = 0 (0%)	N/A
**Stapes surgery**	n = 0 (0%)	n = 2 (5.9%)	*p* = 0.35
**Chronic ear disease**	n = 4 (6.5%)	n = 2 (5.9%)	*p* = 1.00
**Previous ear surgery**	n = 10 (16.1%)	n = 12 (35.3%)	*p* = 0.16

**Table 3:** Investigations and operative findings according to the two groups. OW: oval window. RW: round window. N/A: not applicable.

		Group I (No PLF Identified) (N = 62)	Group II (PLF Identified) (N = 36)	*p-*Value
**CT Scan**	Normal	n = 30 (48.4%)	n = 22 (61.1%)	*p* = 0.23
	Pneumolabyrinth	n = 0 (0%)	n = 2 (5.6%)	
	N/A	n = 32 (51.6%)	n = 12 (33.3%)	
**MRI**	Normal	n = 36 (58%)	n = 22 (61.1%)	*p* = 0.74
	Pneumolabyrinth	n = 2 (3.3%)	n = 0 (0%)	
	N/A	n = 24 (38.7%)	n = 14 (38.9%)	
**VNG**	% of paretic case	53%	36%	*p* = 0.55
	Caloric paresis	29.85% ± 26.89	27.18% ± 32.13	
	N/A	n = 28 (45.16%)	n = 14 (38.88%)	
**Fistula site**	PLF OW	n = 0 (0%)	n = 22 (61.1%)	N/A
	PLF RW	n = 0 (0%)	n = 12 (33.3%)	
	PLF OW + RW	n = 0 (0%)	n = 2 (5.6%)	
**Material used for obliteration**	Fat	n = 7 (11.2%)	n = 6 (16.7%)	*p* = 0.55
	Perichondrium	n = 47 (75.8%)	n = 26 (72.2%)	
	Fat + Perichondrium	n = 8 (13%)	n = 4 (11.11%)	
**Windows obliterated**	RW only	n = 0 (0%)	n = 0 (0%)	*p* = 0.13
	OW only	n = 0 (0%)	n = 4 (11.1%)	
	RW + OW	n = 62(100%)	n = 32 (88.9%)	

**Table 4:** Symptomatology of patients before treatment and 1, 6, and 12 months (M) after surgery. *p*-value before and at 12 months: comparison of Groups I and II. N/A: not applicable.

	Group I (No PLF Identified) N = 62				Group II (PLF Identified) N = 36				*p-*Value	
	Before	1 M	6 M	12 M	Before	1 M	6 M	12 M	Before	12 M
**Hearing loss**	51	50	24	8	32	20	16	4	0.13	1.00
**Stable hearing**	24	34	14	6	14	22	14	6	0.09	0.57
**Fluctuating hearing**	34	14	8	2	14	2	2	0	0.28	1.00
**Progressive hearing loss**	16	4	2	0	2	0	0	0	0.09	N/A
**Sudden hearing loss**	14	0	0	0	14	0	0	0	0.22	N/A
**Aural fullness**	40	16	6	4	16	8	4	0	0.17	0.46
**Tinnitus**	52	40	22	10	28	14	16	6	0.41	0.32
**Instability with motion**	36	16	10	2	12	10	2	0	0.09	1.00
**Instability at rest**	24	6	4	0	4	2	2	2	*0.03*	0.40
**Vertigo (in general)**	30	8	4	0	20	2	4	0	0.62	N/A
**Vertigo with motion**	28	8	4	0	20	2	4	0	0.48	N/A
**Vertigo at rest**	24	6	2	0	8	2	2	0	0.23	N/A
**Nausea and/or vomiting**	20	2	2	0	10	6	0	0	0.74	N/A
**Sensation of falling**	1	0	0	0	0	2	0	0	1.00	N/A

**Table 5:** Evolution of symptoms in the Groups I and II.

	Group I (No PLF Identified) (N = 62)					Group II (PLF Identified) (N = 36)				
	Before	1 M	6 M	12 M	*p* Value	Before	1 M	6 M	12 M	*p* Value
**Tinnitus**	52	40	22	10	0.17	28	14	16	6	0.05
**Hypoacusis**	51	50	24	8	0.005	32	20	16	4	0.009
**Fluctuating hearing**	34	14	8	2	0.003	14	2	2	0	0.001
**Sensation of falling**	2	0	0	0	0.26	0	2	0	0	N/A
**Vertigo (in general)**	30	8	4	0	0.0006	20	2	4	0	0.001
**Aural fullness**	40	16	6	4	0.004	16	8	4	0	0.01

## Data Availability

Data are available upon request due to restrictions (ethical).

## References

[1] de Wit D., Spoor A. (1957). The Gellé test, quantitatively performed, as a measure for the mobility of the stapes. Pract. Oto-Rhinolaryngol..

[2] Hennebert C. (1905). Labyrinthite double reflexe moteur otooculaire. Clinique.

[3] Hornibrook J. (2012). Perilymph Fistula: Fifty Years of Controversy. ISRN Otolaryngol..

[4] Fee G.A. (1968). Traumatic perilymph fistulas. Arch. Otolaryngol..

[5] Stroud M.H., Calceterra T.C. (1970). Spontaneous perilymph fistulas. Laryngoscope.

[6] Ikezono T., Shindo S., Sekiguchi S., Morizane T., Pawankar R., Watanabe A., Miura M., Yagi T. (2009). The Performance of Cochlin-Tomoprotein Detection Test in the Diagnosis of Perilymphatic Fistula. Audiol. Neurotol..

[7] Bhatia N., Lehrer J.F. (2012). Perilymphatic Fistula: An approach to diagnosis and management that provides surer diagnosis and provides medical and surgical management options: Report of six illustrative recent cases. Int. Tinnitus J..

[8] Alzahrani M., Fadous R., Dufour J.J., Saliba I. (2015). Perilymphatic fistulas: Can. we predict the diagnosis?. Eur. Arch. Otorhinolaryngol..

[9] Maitland C.G. (1994). Perilymphatic fistula. Otolaryngol. Clin. North. Am..

[10] Reilly J.S. (1989). Congenital perilymphatic fistula: A prospective study in infants and children. Laryngoscope.

[11] Goodhill V. (1971). Sudden deafness and round window rupture. Laryngoscope.

[12] Fitzgerald D., Getson P., Brasseux C. (1997). Perilymphatic Fistula: A Washington, DC, Experience. Ann. Otol. Rhinol. Laryngol..

[13] Seltzer S., Mccabe B.F. (1986). Perilymph fistula: The iowa experience. Laryngoscope.

[14] Heilen S., Lang C.P., Warnecke A., Lenarz T., Durisin M. (2020). Exploratory tympanotomy in sudden sensorineural hearing loss for the identification of a perilymphatic fistula—Retrospective analysis and review of the literature. J. Laryngol. Otol..

[15] Matsuda H., Hornibrook J., Ikezono T. (2023). Assessing the efficacy of perilymphatic fistula repair surgery in alleviating vestibular symptoms and associated auditory impairments. Front. Neurol..

[16] Smith C.M., Curthoys I.S., Mukherjee P., Wong C., Laitman J.T. (2021). Three-dimensional visualization of the human membranous labyrinth: The membrana limitans and its role in vestibular form. Anat. Rec..

[17] Hornibrook J. (2018). The postural and cognitive Disabilities of chronic perilymph fistula (PLF) after mild head trauma. Annals Clin. Case Rep..

[18] Pelosi S., Schuster D., Jacobson G.P., Carlson M.L., Haynes D.S., Bennett M.L., Rivas A., Wanna G.B. (2013). Clinical characteristics associated with isolated unilateral utricular dysfunction. Am. J. Otolaryngol..

[19] Kohut R.I., Haenel J.L., Waldorf R.A., Thompson J.N. (1979). Minute Perilymph Fistulas: Vertigo and Hennebert’s Sign without Hearing Loss. Ann. Otol. Rhinol. Laryngol..

[20] Nomura Y. (1994). Perilymph Fistula: Concept, Diagnosis and Management. Acta Oto-Laryngologica.

[21] Fraser J.G., Flood L.M. (1982). An audiometric test for perilymph fistula. J. Laryngol. Otol..

[22] Modugno G.C., Magnani G., Brandolini C., Savastio G., Pirodda A. (2006). Could vestibular evoked myogenic potentials (VEMPs) also be useful in the diagnosis of perilymphatic fistula?. Eur. Arch. Otorhinolaryngol..

[23] Campbell K.C., Abbas P.J. (1993). Electrocochleography with postural changes in perilymphatic fistula and Menière’s disease: Case reports. J. Am. Acad. Audiol..

[24] Arts H.A., Adams M.E., Telian S.A., El-Kashlan H., Kileny P.R. (2009). Reversible Electrocochleographic Abnormalities in Superior Canal Dehiscence. Otol. Neurotol..

[25] Morris M.S., Kil J., Carvlin M.J. (1993). Magnetic resonance imaging of perilymphatic fistula. Laryngoscope.

[26] Davidson H.C. (2004). Imaging of the temporal bone. Neuroimaging Clin. N. Am..

[27] Dubrulle F., Chaton V., Risoud M., Farah H., Charley Q., Vincent C. (2020). The round window sign: A sensitive sign to detect peri-lymphatic fistulae on delayed postcontrast 3D-FLAIR sequence. Eur. Radiol..

[28] Venkatasamy A., Al Ohraini Z., Karol A., Karch-Georges A., Riehm S., Rohmer D., Charpiot A., Veillon F. (2020). CT and MRI for the diagnosis of perilymphatic fistula: A study of 17 surgically confirmed patients. Eur. Arch. Oto-Rhino-Laryngol..

[29] Counter S.A., Zou J., Bjelke B., Klason T. (2003). 3D MRI of the in vivo vestibulo-cochlea labyrinth during Gd-DTPA-BMA uptake. NeuroReport.

[30] Zou J., Pyykkö I. (2015). Enhanced oval window and blocked round window passages for middle-inner ear transportation of gado-linium in guinea pigs with a perforated round window membrane. Eur. Arch. Otorhinolaryngol..

[31] Bussières R., Portmann D., Noyon P. (2003). When to suspect a perilymphatic fistula?. Rev. Laryngol. Otol. Rhinol. (Bord).

[32] Sarna B., Abouzari M., Merna C., Jamshidi S., Saber T., Djalilian H.R. (2020). Perilymphatic Fistula: A Review of Classification, Etiology, Diagnosis, and Treatment. Front. Neurol..

[33] Podoshin L., Fradis M., Ben-David J., Berger S.I., Feiglin H. (1994). Perilymphatic fistula—The value of diagnostic tests. J. Laryngol. Otol..

[34] Schneider D., Hahn A., Claussen C.F. (1991). Cranio-corpo-graphy. A neurootological screening test. Acta Otorhinolaryngol. Belg..

[35] Voorhees R.L. (1989). The Role of Dynamic Posturography in Neurotologic Evaluation. Laryngoscope.

[36] Aksoy F., Yenigun A., Senturk E., Tugrul S., Eren S.B., Ozturan O. (2020). Use of topical fluorescein in the diagnosis and localization of perilymphatic fistula. Am. J. Otolaryngol..

[37] Gadre A.K., Edwards I.R., Baker V.M., Roof C.R. (2020). Membranous or Hypermobile Stapes Footplate: A New Anatomic Site Re-sulting in Third Window Syndrome. Front. Neurol..

